# Birth size and the serum level of biological age markers in men

**DOI:** 10.1038/s41598-023-41065-w

**Published:** 2023-08-30

**Authors:** Agnieszka Żelaźniewicz, Judyta Nowak-Kornicka, Bogusław Pawłowski

**Affiliations:** Department of Human Biology, University of Wrocław, Ul. Przybyszewskiego 63, 51-148 Wrocław, Poland

**Keywords:** Ageing, Intrauterine growth

## Abstract

Previous studies showed that intrauterine growth restrictions, resulting in smaller body size at birth, are associated with altered development and the risk of age-related diseases in adult life. Thus, prenatal development may predict aging trajectories in humans. The study aimed to verify if body size at birth is related to biological age in adult men. The study sample consisted of 159 healthy, non-smoking men with a mean age of 35.24 (SD 3.44) years. Birth weight and length were taken from medical records. The ponderal index at birth was calculated. Biological age was evaluated based on serum levels of s-Klotho, hsCRP, DHEA/S, and oxidative stress markers. Pregnancy age at birth, lifestyle, weight, cortisol, and testosterone levels were controlled. The results showed no relationship between birth size and s-Klotho, DHEA/S level, inflammation, or oxidative stress. Also, men born as small-for-gestational-age (N = 49) and men born as appropriate-for-gestational-age (N = 110) did not differ in terms of biological age markers levels. The results were similar when controlled for pregnancy week at birth, chronological age, BMI, testosterone, or cortisol level. The results suggest that there is no relationship between intrauterine growth and biomarkers of aging in men aged 30–45 years from the affluent population.

## Introduction

A large body of evidence links impaired fetal growth and smaller body size at birth with altered development and worsened adult health. The majority of the research focuses on the negative relationship between birth size and the risk of cardio-metabolic diseases and their physiological forerunners, such as hypertension, dyslipidemia, and impaired glycemia^1–3^. There are also studies showing the relationship between lowered birth weight and altered gonadal development^4, 5^, earlier age at menarche^6^, and higher risk of reproductive disorders^5, 7^. Furthermore, birth weight is positively associated with postnatal growth and adult height^8^, and negatively with adult obesity risk^9^. The observed effects extend across the normal range of birth weight, have been described in populations of different ages, sex, and ethnic origin, and occur independently of the duration of gestation or adult weight^10^.

It has been suggested that these relationships result from programming, a process whereby a stimulus or insult at a critical period in development results in permanent adaptation of the organism’s structure and physiology affecting its development across ontogenesis^11^. The mechanism underlying these adaptations may result from the altered functioning of the key endocrine axes that condition life history (LH) trajectories, such as the hypothalamic–pituitary–adrenal axis, hypothalamic–pituitary–gonadal axis, and growth hormone-insulin-like growth factor axis in small for gestational age (SGA) children, resulting in alterations in age at menarche, growth, or age at onset of cardio-metabolic diseases^12–14^. Thus, early-life exposures seem to impact a range of systems that are crucial for LH pace and trade-offs, which often have hormonal underpinnings, and the relationship between birth size and the risk of chronic disease may result from the altered pace of aging^15^.

Across the lifespan, the consequences of individual differences in genetic endowment, cellular biology, hormonal axes functioning, and life experience accumulate, driving the divergence of biological age from chronological age for some people^16, 17^. As a result, there is a marked variation in the rate of an individual’s biological aging and the age when we experience chronic diseases (e.g. cardio-metabolic diseases) and declining capacities (e.g., reduced strength, cognitive decline)^18–20^. Also, birth size may be linked with biological age in healthy adult individuals which in consequence may lead to various risks of chronic disease development. Recent research reported a negative relationship between birth weight and DNA methylation in men but not in women of 20.8–22.5 years^21^ or in epigenetic age acceleration over the first 3 years of life^22^. Another study showed contradictory results for various DNA methylation clocks in young and middle-aged adults^23^ or no relationship between birth weight and telomere length^24^. Including other than DNA-based measures of biological age in the studies on early developmental origins of aging trajectories may help to understand these contradictory results.

There are many physiological markers of biological age, including specific proteins, hormones, oxidative stress, or inflammation level. One such specific protein is α-Klotho whose level is positively associated with slowing the aging process and longevity^25, 26^ and negatively with the risk of neurodegenerative disease^27^, hypertension^28^, triglycerides levels^29^, and chronic inflammation^30^.

Among the hormonal markers of biological age, dehydroepiandrosterone (DHEA) and its sulfated metabolite (DHEA-S) are one of the most important. Concentrations of these hormones in humans typically decrease steadily with age, although showing inter-individual variability, approaching a nadir (c.a. 20% of the peak concentration) at approximately 65–70 years, the age at which many diseases of aging become markedly more prevalent^31, 32^. Although it is suggested that the main role of DHEA/S is due to being a precursor of sex hormones, some studies suggest that DHEA/S per se may have preventive and therapeutic properties against many age-associated diseases^33–35^ and their levels correlate positively with muscle mass and strength^36^, bone mineral density^37^, skin condition^38^, cognitive functioning^39^, cardiovascular health^40^, glucose tolerance^41^, lower oxidative stress^42^ and the risk of autoimmune disease^43^.

Another physiological marker of biological age is oxidative stress level. Free radicals are produced in the mitochondrial respiratory chain, an oxidative burst of neutrophils, macrophages, dendritic cells, and monocytes, or as a response to environmental factors^44^. Their negative effects are neutralized by antioxidant defenses, and when the antioxidant defenses are reduced, oxidative stress occurs resulting in oxidative damage to various cell molecules. This damage increases and accumulates with age causing progressive damage and functional decline^45, 46^. Oxidative stress is involved in several age-related conditions, such as cardiovascular disease^47^, neurodegenerative diseases^48^, diabetes^49^, cancer^50^, and also is related to shorter lifespan^51^.

Among a wide range of oxidative stress markers, protein carbonyls (PCs) are one of the key products of protein oxidation in cells and tissues. Elevated PCs level has been detected in elders and several aging-related diseases including neurodegenerative and cardiovascular diseases, cancer, and diabetes^52–54^. Similarly, 8-epi-prostaglandin F2α (8-epi-PGF2α) is a major F2-isoprostane formed during lipid oxidation and a reliable marker of oxidative stress^55^. Elevated levels of 8-epi-PGF2α correlate positively with glucose levels, HOMA-IR index value, and risk of diabetes, hypertension, dyslipidemia, and cancer^56, 57^. Furthermore, 8-hydroxy-2′-deoxyguanosine (8-OHdG) is the most commonly observed ROS-induced oxidative DNA lesion that may induce mutations in replicating DNA and is a biomarker of oxidative DNA damage^58^. 8-OHdG level is often used as a marker of age-related oxidative damage accumulation^59–61^ linked with increased risk of carcinogenesis^62^, metabolic syndrome^63^, or neurodegenerative diseases^64^.

Aging is also associated with immune dysfunction that coexists with an upregulation of the inflammatory response that occurs with age, resulting in a low-grade chronic systemic proinflammatory state. The latter is illustrated by a two- to four-fold increase in the levels of C-reactive protein (CRP) or interleukin (IL)-6^65^, which can be also perceived as markers of biological age. A chronic inflammatory state is involved in the pathogenesis of most age-associated diseases^66, 67^, a robust risk predictor for cardiovascular^68^, neurodegenerative^69^, autoimmune diseases^70^, osteoporosis^65^, and cancer^71^.

Thus, the study aims to verify if the birth size is related to biological age as measured based on s-Klotho, adrenal hormones (DHEA/S), oxidative stress (PCs, 8-epi-PGF2α, RNA/DNA damage markers—8-OHdG and 8-OHG), and inflammation levels (hsCRP).

## Results

### Descriptive statistics

Descriptive statistics are presented in Table 1.

**Table 1:** Descriptive statistics (N = 159).

	M	SD	Min	Max
Age (years)	35.24	3.42	29.83	44.29
Birth weight (g)	3523.15	440.64	2600.00	5000.00
Ponderal index (kg/m^3^)	2.15	0.30	1.45	2.94
Pregnancy week at birth (week)	38.97	1.54	35.00	44.00
s-Klotho level (pg/ml)	1158.46	470.89	306.70	2686.50
DHEA (ng/ml)	9.07	4.79	1.15	25.00
DHEA-S [µg/ml]	4.00	1.00	0.94	6.28
hsCRP (µg/ml)	1.22	1.29	0.01	6.49
8-Isoepiprostaglandine (pg/ml)	206.84	66.50	97.53	383.53
RNA/DNA (pg/ml)	16,845.05	6141.24	6209.10	37,793.63
Protein carbonyls (ng/ml)	32.67	15.54	2.85	125.11
Composite measure of oxidative stress	0.00	0.56	− 2.31	0.99
Composite measure of biological age	0.00	0.42	− 1.44	1.39
TAC (µm)^a^	2.00	0.57	0.97	3.20
Body adiposity (%)	22.94	6.76	7.07	42.30
Cortisol (ng/ml)	323.55	60.70	149.00	488.73
Testosterone (ng/dl)	493.19	180.37	146.90	1157.00

^a^Serum total antioxidant capacity.

Men who practiced sport had a lower level of hsCRP (M = 1.05, SD = 1.21) compared to men who did not practice sport (M = 1.42, SD = 1.35) (t(157) = − 2.09, *p* = 0.04, Cohen’s d = 0.33). Men who practiced sport had higher testosterone level (M = 518.98, SD = 171.61) compared to men who did not practice sport (M = 462.81, SD = 186.81) (t(157) = 1.97, *p* = 0.05, Cohen’s d = − 0.31). The two groups did not differ in terms of the other markers of biological age or chronological age (in each case *p* > 0.23). Men who smoked in the past and men who have never smoked did not differ in terms of markers of biological age (in each case *p* > 0.24). S-Klotho level was lower in men who drank alcohol more often (F(2,156) = 5.06, *p* = 0.007, η^2^ = 0.06). These three groups did not differ in terms of other markers of biological age (*p* > 0.11).

DHEA, DHEAS-S levels, and the composite index of biological age were negatively correlated with age. hsCRP was positively related to adiposity and the composite index of biological age was negatively related to adiposity. DHEA/S and composite index of biological age were also positively related to cortisol levels. hsCRP correlated negatively with testosterone levels (Table 2).

**Table 2:** The relationship between markers of biological age and controlled variables (N = 159).

	Age (year)		TAC (µm)^c^		Adiposity (%)		Cortisol (ng/ml)		Testosterone (ng/dl)	
	R	*P*	r	*p*	r	*p*	r	*p*	r	*p*
LOG Klotho (pg/ml)	0.07	0.35	0.01	0.88	0.03	0.70	0.02	0.80	0.05	0.50
LOG DHEA (ng/ml)	− **0.24**	**0.002**	0.04	0.61	− 0.15	0.05	**0.53**	** < 0.001**	0.13	0.10
DHEA-S (µg/ml)	− **0.22**	**0.005**	0.01	0.92	0.004	0.96	**0.15**	**0.05**	− 0.02	0.83
LOG hsCRP (µg/ml)	0.05	0.50	0.05	0.54	**0.47**	** < 0.001**	− 0.16	0.04	− **0.16**	**0.04**
OS^a^	0.03	0.68	0.01	0.90	− 0.06	0.44	− 0.06	0.48	0.002	0.98
Biological age^b^	− **0.18**	**0.02**	− 0.003	0.97	− **0.21**	**0.008**	**0.32**	** < 0.001**	0.09	0.27

Significant values are in bold.

^a^Composite measure of oxidative stress.

^b^Composite measure of biological age.

^c^Serum total antioxidant capacity.

Birthweight was positively related to pregnancy week at birth and TAC levels and negatively with cortisol level. The ponderal index was not related to any controlled variable (Table 3).

**Table 3:** The relationship between measures of birth size and controlled variables (N = 159).

	Birthweight (g)		Ponderal index (kg/m^3^)	
	r	*p*	r	*p*
Age (year)	− 0.01	0.90	0.08	0.33
Pregnancy week at birth	**0.29**	**< 0.001**	0.09	0.27
TAC (µm)^a^	**0.17**	**0.03**	0.08	0.31
Adiposity (%)	0.09	0.25	0.06	0.49
Cortisol (ng/ml)	− **0.17**	**0.04**	− 0.08	0.33
Testosterone (ng/dl)	− 0.0	0.37	− 0.08	0.30

Significant values are in bold.

^a^Serum total antioxidant capacity.

### The relationship between birth size and markers of biological age

The results of zero-order correlations showed that the s-Klotho level showed no relationship between birth weight or ponderal index and markers of biological age (Table 4). Also, there was no difference between men classified as SGA (N = 49) and classified as AGA (N = 119) comparing the mean values of the levels of markers of biological age (in each case *p* > 0.28).

**Table 4:** The results of the correlation analyses between birth size and markers of biological age (N = 159).

	Birthweight (g)		Ponderal index (kg/m^3^)	
	r	*p*	r	*p*
LOG s-Klotho (pg/ml)	0.05	0.57	− 0.04	0.66
LOG DHEA (ng/ml)	− 0.10	0.23	− 0.05	0.56
DHEA-S (µg/ml)	− 0.07	0.39	0.03	0.72
LOG hsCRP (µg/ml)	0.09	0.28	0.09	0.73
Oxidative stress^a^	− 0.02	0.83	− 0.07	0.36
Biological age^b^	− 0.08	0.29	− 0.05	0.57

^a^Composite measure of oxidative stress.

^b^Composite measure of biological age.

### The relationship between birth size and biological age parameters controlled for cofounders

S-Klotho level was not related to birth weight or ponderal index when controlled for pregnancy week at birth and frequency of the alcohol drinking. S-Klotho level was only related to the frequency of alcohol drinking (Table 5). There was no difference in the mean s-Klotho levels between men from the three terciles of birthweight (F(2,156) = 0.04, *p* = 0.96, η^2^ < 0.001) or ponderal index (F(2,156) = 0.23,* p* = 0.80, η^2^ = 0.003).

**Table 5:** The results of regression analyses for the relationship between birth weight (Model 1) or ponderal index (Model 2) and s-Klotho level (log) controlled for cofounders (N = 159).

	Β	SE(β)	t(155)	*p*
Model 1: F(3,155) = 3.91, *adj. R*^*2*^ = 0.05, *p* = 0.01, 1 − β = 0.83				
Birthweight (g)	0.01	0.08	0.13	0.90
Pregnancy week at birth	0.10	0.08	1.22	0.23
Freq. of alcohol drinking	− **0.24**	**0.08**	− **3.12**	**0.002**
Model 2: F(3,155) = 3.95, *adj. R*^*2*^ = 0.05, *p* = 0.01, 1 − β = 0.83				
Ponderal index (kg/m^3^)	− 0.03	0.08	− 0.33	0.74
Pregnancy week at birth	0.10	0.08	1.33	0.18
Freq. of alcohol drinking	− **0.24**	**0.08**	− **3.09**	**0.002**

Significant values are in bold.

DHEA level was not related to birth weight or ponderal index when controlled for pregnancy week at birth, age, adiposity, and cortisol level (factors that may impact DHEA levels—Table 2). DHEA was positively related to cortisol levels (Model 1 and 2—Table 6). Similarly, there was no relationship between DHEA-S and birth weight or ponderal index when controlled for pregnancy week at birth, chronological age, and cortisol level. DHEA-S was negatively related to age (Model 3 and 4—Table 6). There was no difference in the mean DHEA levels between men from the three terciles of birthweight (F(2,156) = 0.71, *p* = 0.49, η^2^ = 0.01) or ponderal index (F(2,156) = 0.12, *p* = 0.88, η^2^ = 0.001). There was also no difference in the mean DHEA-S levels between men from the three terciles of birthweight (F(2,156) = 0.48, *p* = 0.62, η^2^ = 0.006) or ponderal index (F(2,156) = 0.30, *p* = 0.74, η^2^ = 0.004).

**Table 6:** The results of multiple regression analyses for the relationship between DHEA/S levels and birth weight (Model 1 and 3) or ponderal index (Model 2 and 4) controlled for cofounders (N = 159).

	Β	SE(β)	t(153)	*p*
Model 1 (Dependent variable—LOG DHEA [ng/ml]): F(5, 153) = 13.63, *adj. R*^*2*^ = 0.29, *p* < 0.001, 1 − β = 0.99				
Birthweight (g)	− 0.002	0.07	− 0.04	0.97
Age (years)	− **0.15**	**0.07**	− **2.25**	**0.03**
Adiposity (%)	− 0.08	0.07	− 1.24	0.22
Cortisol (ng/ml)	**0.49**	**0.07**	**6.91**	** < 0.001**
Pregnancy week at birth	− 0.02	0.07	− 0.26	0.80
Model 2 (Dependent variable—LOG DHEA [ng/ml]): F(5, 153) = 13.64, adj. R^2^ = 0.29, *p* < 0.001, 1 − β = 0.99				
Ponderal index (kg/m^3^)	0.01	0.07	0.15	0.88
Age (years)	− **0.16**	**0.07**	− **2.26**	**0.03**
Adiposity (%)	− 0.09	0.07	− 1.25	0.21
Cortisol (ng/ml)	**0.49**	**0.07**	**7.03**	** < 0.001**
Pregnancy week at birth	− 0.02	0.07	− 0.29	0.77
Model 3 (Dependent variable—DHEA-S [µg/ml]): F(4, 154) = 2.70, adj. R^2^ = 0.04, *p* = 0.03, 1 − β = 0.71				
Birthweight (g)	− 0.04	0.08	− 0.51	0.61
Age (years)	− **0.20**	**0.08**	− **2.51**	**0.01**
Cortisol (ng/ml)	0.11	0.08	1.40	0.16
Pregnancy week at birth	− 0.03	0.08	− 0.38	0.70
Model 3 (Dependent variable—DHEA-S [µg/ml]): F(4, 154) = 2.77, adj. R^2^ = 0.04, *p* = 0.03, 1 − β = 0.71				
Ponderal index (kg/m^3^)	0.06	0.08	0.74	0.46
Age (years)	− **0.20**	**0.08**	− **2.54**	**0.01**
Cortisol (ng/ml)	0.13	0.08	1.58	0.12
Pregnancy week at birth	− 0.05	0.08	− 0.62	0.53

Significant values are in bold.

hsCRP level was not related to birth weight or ponderal index when controlled for pregnancy week at birth, adiposity and regular physical activity. Inflammation was positively related only to adiposity level (Model 1 and 2—Table 7). We have also run similar model including also testosterone and cortisol and we also found no relationship between birth size and hsCRP level and the model with higher number of predictors had worse fit (BIC = 273.181 vs BIC = 265.72). Additionally, we verified if adiposity moderated the relationship between birth weight/ponderal index and hsCRP by introducing the interaction between birthweight and body adiposity in the model. The model showed no relationship between birth weight and adiposity (*p* = 0.13 for birth weight; *p* = 0.94 for ponderal index). There was no difference in the mean inflammation levels between men from the three terciles of birthweight (F(2,156) = 0.47, *p* = 0.63, η^2^ = 0.006) or ponderal index (F(2,156) = 0.31, *p* = 0.73, η^2^ = 0.006).

**Table 7:** The results of multiple regression analyses for the relationship between hsCRP level and birth weight (Model 1) or ponderal index (Model 2) controlled for cofounders (N = 159).

	Β	SE(β)	t(154)	*p*
Model 1 (Dependent variable—LOG hsCRP): F(4, 154) = 11.62, *adj. R*^*2*^ = 0.21, *p* < 0.001, 1 − β = 0.99				
Birthweight (g)	0.05	0.07	0.65	0.51
Pregnancy week at birth	− 0.03	0.07	− 0.41	0.68
Adiposity (%)	**0.45**	**0.07**	**6.31**	**< 0.001**
Regular physical activity (y/n)^a^	− 0.09	0.07	− 1.21	0.23
Model 2 (Dependent variable—LOG hsCRP): F(4, 154) = 11.48, adj. R^2^ = 0.21, *p* < 0.001, 1 − β = 0.99				
Ponderal index (kg/m^3^)	0.001	0.07	0.01	0.99
Pregnancy week at birth	− 0.02	0.07	− 0.24	0.81
Adiposity (%)	**0.46**	**0.07**	**6.35**	**< 0.001**
Regular physical activity (y/n)^a^	− 0.09	0.07	− 1.22	0.22

Significant values are in bold.

^a^Coded as 0 = ”no” and 1 = ”yes”.

OS level was not related to birth weight or ponderal index when controlled for pregnancy week at birth and TAC (in order to account for a possible impact of antioxidant capacity on the relationship between birth size and oxidative stress level (Table 8). There was no difference in the mean OS levels between men from the three terciles of birthweight (F(2,156) = 0.17, p = 0.85, η^2^ = 0.002) or ponderal index (F(2,156) = 0.91, p = 0.41, η^2^ = 0.01).

**Table 8:** The results of multiple regression analyses for the relationship between oxidative stress levels and birth weight or ponderal index controlled for cofounders (N = 159).

	Β	SE(β)	t(156)	*p*
Model 1 (Dependent variable—OS level): F(3,155) = 0.35, adj. R^2^ < 0.001, *p* = 0.79, 1 − β = 0.05				
Birthweight (g)	− 0.01	0.08	− 0.07	0.94
Pregnancy week at birth	0.09	0.08	1.00	0.32
TAC (µm)^a^	− 0.01	0.08	− 0.11	0.91
Model 2 (Dependent variable—OS level): F(3,155) = 0.59, adj. R^2^ < 0.001, *p* = 0.62, 1 − β = 0.05				
Ponderal index (kg/m^3^)	0.07	0.08	0.85	0.40
Pregnancy week at birth	0.08	0.08	0.96	0.34
Freq. of alcohol drinking	− 0.01	0.08	− 0.17	0.87

^a^Serum total antioxidant capacity.

The composite index of biological age was also not related to birth weight or ponderal index when controlled for chronological age, adiposity, and cortisol level. Biological age was negatively related to adiposity level and positively to cortisol level (Table 9). There was no difference in the mean biological age index between men from the three terciles of birthweight (F(2,156) = 0.76, p = 0.47, η^2^ = 0.01) or ponderal index (F(2,156) = 0.29, p = 0.40, η^2^ = 0.004).

**Table 9:** The results of multiple regression analyses for the relationship between biological age index and birth weight (Model 1) or ponderal index (Model 2) controlled for cofounders (N = 159).

	Β	SE(β)	t(153)	*p*
Model 1 (Dependent variable—biological age): F(5, 153) = 5.54, adj. R^2^ = 0.13, *p* < 0.001, 1 − β = 0.97				
Birthweight (g)	− 0.01	0.08	− 0.12	0.90
Pregnancy week at birth	− 0.05	0.08	− 0.69	0.49
Adiposity (%)	− **0.17**	**0.08**	− **2.23**	**0.03**
Cortisol (ng/ml)	**0.27**	**0.08**	**3.50**	**< 0.001**
Age (years)	− 0.14	0.08	− 1.83	0.07
Model 2 (Dependent variable—biological age): F(5, 153) = 5.54, adj. R^2^ = 0.13, *p* < 0.001, 1 − β = 0.97				
Ponderal index (kg/m^3^)	0.001	0.08	0.02	0.99
Pregnancy week at birth	− 0.06	0.08	− 0.76	0.45
Adiposity (%)	− **0.17**	**0.08**	− **2.23**	**0.03**
Cortisol (ng/ml)	**0.27**	**0.08**	**3.57**	**< 0.001**
Age (years)	− 0.14	0.08	− 1.82	0.07

Significant values are in bold.

## Discussion

The results of this study showed no relationship between birth size and physiological markers of biological age in men between 30 and 45 years. Birth weight but not the ponderal index was only positively related to TAC level and negatively to cortisol level. Furthermore, the levels of biological age markers were mainly related to lifestyle factors, adiposity, and testosterone level. hsCRP was negatively related to physical activity and testosterone level and positively to adiposity. The S-Klotho level was negatively related to the frequency of alcohol drinking. DHEA and DHEA-s were negatively related to age and positively to cortisol levels.

The results of the previous research on the relationship between birth size and senescence showed contradictory results. Animal studies have shown that growth restriction during pregnancy and subsequent catch-up growth diminishes telomere length in rats that ultimately have also a reduced life span^72–75^. In human adults with a mean age of 43.04 years, birth weight was inversely associated with p21 gene expression but not with p16 expression, markers of cellular senescence. What is interesting, the expression of p21 but not p16, was positively correlated with BMI gain at 29 years and current BMI^76^. In men, lower birth weight predicted advanced biological aging based on epigenetic clocks^21^. Furthermore, no association was found between birth weight and telomere length in humans^24, 77, 78^. Masterson et al.^79^ showed that heavier birth weight was associated with longer telomere length but the relationship was attenuated by maternal age at birth and probably rather related to childhood growth. Other studies show that BMI in childhood may be more important for senescence than birth size^80, 81^. In this study, no relationship between birthweight and biological age was found as measured as a composite index of physiological markers of biological age.

We also found no relation between birth weight and inflammation level, also when body adiposity was controlled. This result is in line with sparse studies showing no relationship between birth size and inflammation^82^ and at variance with previous studies which showed that birth weight was negatively associated with adolescent or adult CRP levels^83–85^. However, previous research has also shown that this relationship may be mediated by an elevated body fat mass among individuals with lower birthweight^85–87^. Birth weight-adult inflammation risk is mediated by later BMI and BMI change and inflammatory risk related to birthweight can be offset by weight loss^88^. However, in our study, although hsCRP level was positively related to body adiposity, physical activity, and testosterone level, these factors did not moderate the relationship between birth weight and inflammation level. Other studies suggest that breastfeeding^89^, life-course socioeconomic status^90^, and diet^91^ are important factors in moderating the relationship between birth weight and adult inflammation. Also, the role of such modifiers as sex or age is still unclear^92^. We cannot exclude the possibility that some lifestyle factors that were not controlled in our study might impact the relationship between inflammation and birth size.

We found no relationship between birth size and s-Klotho level in adult men. Previous research has shown that newborns with SGA have lower levels of klotho^93^. No research, so far investigated if the relationship persists to adulthood. What is interesting, the s-Klotho level was also not related to physiological markers of cardiometabolic risk in this group^94^. This is in line with other research showing no relationship between s-Klotho level and health outcomes^95–97^. It is possible that the role of s-Klotho as a marker of biological age may depend on age and may be more valid in older individuals^94, 98^ Also, other research suggested sex differences in the relationship between the s-Klotho level and health components^99^ that might be explained by different profiles of sex hormones and the interaction between s-Klotho and testosterone or estradiol levels^100, 101^. However, the physiological effects of sex steroid hormones on klotho levels have not been completely elucidated and require further studies. Furthermore, the only factor related to klotho level was the frequency of alcohol drinking which is in line with the previous research^102–104^. Quintero-Platt et al.^105^ observed higher s-Klotho levels among alcoholics than in controls, but these differences were dependent on the presence of cirrhosis. This higher s-Klotho plasma levels in cirrhotic patients could be due to a pathologic status, where the liver function is impaired, and thus not applicable to healthy individuals. Several studies showed an increase in s-Klotho plasma levels in diabetics^106^, patients with acromegaly^107^, and patients with autosomal dominant polycystic kidney disease^108^. However, it is also important to note that many studies show a positive correlation between s-Klotho and markers of health and biological age^109, 110^, also in patients with various disorders^111^.

We also found no relationship between size at birth and DHEA/S level in adult men. Most of the research on the relationship between birth size and adrenal androgens level was conducted in children or young adults and the results are equivocal. For instance, previous research showed a relationship between birth size and DHEA and DHEAS levels in women but not in men of age 18–22 years^112^ On the other hand, higher DHEAS levels at 7 years old were also associated with lower birth weight and higher adiposity^113^. Although, experimental data in animals and recent human observations have suggested that an alteration in the set point of the hypothalamic–pituitary–adrenal axis entails important long-term change that occurs in association with reduced fetal growth^33^ we did not observe the relationship between DHEA/S level and birth weight in men of 30–45 years. We only confirmed that DHEA/S level was only inversely related to chronological age and positively to cortisol level.

Many known or suspected causes or conditions associated with adverse (poor or excessive) fetal growth or preterm birth have been associated with oxidative stress (OS). Although OS may be one of the mechanisms linking impaired fetal growth with certain chronic diseases in adulthood^114, 115^ we found no relationship between birth size and OS level in men of 30–45 years old. Previous research showed increases in oxidative stress markers in SGA children^116–118^ and male rats exposed to maternal protein restrictions^119^ or placental insufficiency^116^. The mechanisms of oxidative stress programming may be through directly modulating gene expression or indirectly through the effects of certain oxidized molecules, which could be compensated by increased antioxidant levels^114^. Interestingly, we found a positive relationship between birth weight, but not ponderal index, and serum total antioxidant capacity. Animal studies suggest that the antioxidant expression and activity are up-regulated in the IUGR (intrauterine growth restriction) rats that are normotensive in young adulthood, implicating a compensatory mechanism that may be protective against the generation of reactive oxygen species in adult IUGR rats^119^. Also, human studies have shown that SGA children have elevated OS markers and also some antioxidants^120^. Possibly, oxidative stress in adults born as small for gestational age can be attenuated by elevated antioxidant levels.

This research study has several methodological strengths. Study subjects were homogeneous in terms of age and ethnic background. Information on birth weight and gestational age at birth was drawn from data recorded by hospital or clinic staff in the maternal and child health handbook. Several methodological limitations of the current study should also be clarified. First, the study had a cross-sectional design, thus we cannot exclude the influence of various factors that could impact the relationship during ontogeny. Second, biological age markers were assayed only once. Despite the rigorous study protocol, we cannot exclude the possibility of some intra-individual variability in the measured markers. Finally, the relatively small sample size may limit the statistical power of the analysis, and small differences in biological age as a function of birth weight may not have been detected.

## Materials and methods

This study was a part of a broader project on factors related to men’s health. All methods were carried out by relevant guidelines and regulations and were reviewed and approved by the Commission of Bioethics at Wroclaw Medical University (nr 222/2019). All men signed written informed consent for participation in the study.

### Participants

Information on the ongoing study was posted in local newspapers, on social media, and broadcasted on the local radio. 209 men (M_age_ = 36.14 ± 3.53) were recruited from an urban Polish population after the initial screening to exclude chronic and acute health problems. Information on birth parameters was available for 183 men. From this sample, 24 men were excluded due to the following reasons: (1) chronic diseases—e.g. diabetes (N = 3); (2) regular smoking (N = 8); (3) CRP > 10 mg/l, indicating ongoing inflammation (N = 1); (4) incomplete measurements of physiological markers of biological age (N = 12). Health status was evaluated based on inflammation, blood morphology, and self-reported health and none of the participants exhibited symptoms of infection. Thus, the final analyses were conducted on 159 healthy men of mean age 35.24 ± 3.42 years (29.83–44.29 years). An a priori power analysis was conducted using G*Power^126^ for minimum sample size estimation. Results indicated the required sample size to achieve 80% power for detecting a medium effect at a significance criterion of α = 0.05 and five predictors is 92 for the regression analysis. Thus, the obtained sample size of N = 159 is adequate to test the study hypothesis.

### Procedures

Participants were asked to refrain from physical activity, heavy meals, and alcohol for 24 h before the study visit. A fasting blood sample was taken between 7:30 a.m. and 9:00 a.m. for blood biochemical and hormone analyses. In addition to serum markers of biological age, also testosterone and cortisol levels were measured. Age-related decline in testosterone levels is linked with increased vulnerability to multiple chronic diseases^121^. We controlled also cortisol level as its level contributes to the pathophysiological mechanisms of aging^122^. Adiposity may impact the relationship between birth weight and measures of the adult biological condition^123^ thus participants’ body adiposity was measured with bioimpedance analysis (SECA mBCA 515).

The participants completed a questionnaire about demographic data, current and past health problems, medications, smoking, and alcohol drinking patterns. Among the participants, 28 individuals declared that they had regularly smoked in the past (quit at least one year before the visit) and 131 stated that they had never smoked. Participants were also asked about the level of physical activity: the number and length (in minutes) of training per week and the type of sport practiced. The types of sports activities practiced by the participants were comparable in terms of intensity (running, biking, swimming, football, basketball, calisthenics, tennis, squash, CrossFit, and strength workout). The participants were divided into two categories: (1) physically active (N = 86), which included individuals who declared regular physical activity at least 60 min/week; and (2) inactive (N = 73) i.e. with no regular sport activity. There were no professional sportsmen in the study sample. Participants were also asked how often they drink alcohol and based on their answers they were divided into three groups: (1) rarely—i.e. once per month or less often (N = 40), (2) sometimes—i.e. 2–4 times per month (N = 72), (3) often—i.e. 2–3 times per week (N = 47).

### Birth parameters

Information on participants’ birth weight and length, and health condition at birth was obtained from the medical records (personal “health books”). The ponderal index at birth was calculated as birth 0.1 × weight/(birth length)^3^ and expressed in kg/m^3^. The ponderal index value below 2.0 indicates asymmetrical intrauterine growth restrictions. We controlled for pregnancy week at birth, as it may impact the birth size also of children born in term^124^.

### Biological age markers measurement

Blood samples were centrifuged and serum was collected and stored at − 80 °C until the analyses. Sample preparation and all test procedures followed the manual supplied with each ELISA kit.

Serum soluble circulating Klotho (s-Klotho) level was measured with enzyme-linked immunosorbent assay (ELISA) using commercial kits (IBL^®^ Code no 27998; with inter-assay and intra-assay coefficient of variation less than 11.4% and less than 3.5% respectively with assay sensitivity of 6.15 pg/ml), following the manufacturer’s protocol. Calibrators (standards supplied with the kit) and serum samples were assayed in duplicate and the average absorbance value was used to calculate hormone concentration. The standard curve was created by plotting mean absorbance values for each standard (Y axis) against its concentration (X axis). Total s-Klotho concentration was calculated in relation to the standard curve, multiplied by the dilution ratio, and expressed in pg/ml.

Serum 8-epi-PGF2α level was assayed with an Elabscience competitive-ELISA kit (cat. No E-EL-0041) with intra- and inter-CV less than 7%. Protein carbonyls were assayed with a MyBioSource ELISA kit (cat. No MBS3802635). DNA/RNA oxidative damage was assayed with a Cayman ELISA kit (cat. No 589320) with inter- and intra- CV less than 12%. The kit allows to measure of DNA/RNA oxidative damage, asses 8-OH-dG from DNA, 8-OHG from RNA, and 8-hydroxyguanine from either DNA or RNA.

Serum hsCRP level was measured using a DEMEDITEC ELISA kit (cat no 740011) with inter- and intra- CV less than 6.3% and 6.9% respectively.

Serum DHEA and DHEA-S levels were assayed using a DEMEDITEC competitive ELISA kit. DHEA (cat no DEH3344) level was measured with inter- and intra-CV less than 6.9% and less than 6.9% respectively. DHEA-S (cat no DEH3366) level was measured with inter- and intra-CV less than 12.2% and less than 6.8% respectively.

Serum total antioxidant capacity (TAC) was assayed with a Cayman ELISA kit (cat. No 709001), with an inter-assay CV is 3% and an intra-assay CV is 3.4%. The kit allows for assessing of aqueous- and lipid-soluble antioxidants, thus the combined antioxidant capacities of all its constituents including vitamins, proteins, lipids, glutathione, uric acid, etc. were assessed.

Serum cortisol level was measured using a DEMEDITEC ELISA kit (cat no DEH3388) with inter- and intra-CV less than 9.2% and less than 7.2% respectively.

Serum samples and calibrators (and controls if supplied with a kit) were assayed in duplicate in all ELISA tests. Absorbance (OD-optical density) was measured using a microplate reader (ASUS UVR340) with at λ recommended by each manual. The calculation of results was based on the calibration curve (for absorbance value for each sample the corresponding concentration from the calibration curve was determined). The average value of sample concentration (from duplicate measures) was used in analyses.

Quantitative measurement of total testosterone was evaluated by a certified analytical laboratory (DIAGNOSTYKA^®^) using a Cobas analyzer and expressed in ng/ml.

### Statistical analyses

Normality was assessed based on Kolmogorov–Smirnov’s tests, kurtosis, skewness, and plot visual inspection. The results of Kolmogorov–Smirnov’s test showed that values of age, birth weight, ponderal index, DHEAS-S, TAC, body adiposity, cortisol, and testosterone levels had a normal distribution. Distribution of pregnancy week at birth was assessed as normal based on the z-scores values for kurtosis and skewness that were below 3.29. S-klotho, DHEA, hsCRP, 8-isoepiprostaglandine, and RNA/DNA levels were log-transformed due to positive results of the Kolmogorov–Smirnov’s test, high kurtosis and/or skewness levels.

An aggregate measure incorporating oxidative stress markers should more accurately assess the levels of antioxidant potential and OS than a single biomarker^125^. Thus, an aggregate measure of oxidative stress was calculated by Z scoring each OS biomarker (each assessing different outcomes of oxidative stress) and averaging for each individual the three Z scores.

Similarly, an aggregate measure of biological age was calculated by Z scoring each biomarker (s-Klotho, hsCRP, DHEA, DHEA-S, 8-isoepiprostaglandine, RNA/DNA, PCs levels) and averaging for each individual the seven Z scores. Z scores of hsCRP and oxidative stress markers were multiplied by − 1 to account for the inverse relationship with biological age. The higher values of this composite measure indicated younger biological age.

Similarly, as in our previous study^94^, the t-test was used to verify if participants who smoked in the past (N = 28) and participants who have never smoked (N = 131) differed in terms of markers of biological age. T-test was also used to verify if participants classified as physically active (N = 86) and participants classified as inactive (N = 73) differed in terms of markers of biological age. ANOVA was used to verify if participants differed in terms of levels of s-Klotho and cardiometabolic risk markers levels depending on how often they drink alcohol: (1) rarely (N = 40); (2) sometimes (N = 72); (3) often (N = 47). Tukey test was used as a post-hoc test. Pearson correlation was to verify if birth weight and ponderal index are related to controlled variables (chronological age, adiposity, cortisol, testosterone).

First, the relationship between birth parameters and biological age markers levels was verified with Pearson correlation analysis. The mean levels of markers of biological age were compared between men classified as small-for-gestational-age (SGA; N = 49) and men classified as appropriate for gestational age (AGA; N = 110) using a T-test. Participants were divided into three groups according to the values of terciles of birth weight: (1) the first tercile (BW ≤ 3300; N = 53); (2) the 2nd tercile (3300 < BW < 3700; N = 52); (3) the 3rd tercile (BW ≥ 3700; N = 54). Participants were also divided into three groups according to the values of terciles of ponderal index: (1) the first tercile (PI ≤ 2.024; N = 53); (2) the 2nd tercile (2.02 < PI ≤ 2.2, N = 53); (3) the 3rd tercile (PI > 2.26; N = 53). ANOVA was used to compare the differences between the three groups divided according to terciles of birth weight and ponderal index in terms of markers of biological age. Then, a simple linear regression model was used to test the association between levels of biological age markers. In each regression analysis, the pregnancy week at birth was controlled as it may impact an individual’s birth size. We controlled also other factors related to the markers of biological age such as chronological age, body adiposity, alcohol use, testosterone and cortisol level, and physical activity. The predictors for each analysis were selected based on the results of the t-test and Pearson correlation analysis for the relationship between biological age markers and potential confounders.

Analyses were performed with Statistica 12.0 software. The results were interpreted as statistically significant if *p* < 0.05.

### Supplementary Information

Supplementary Information.

## Data Availability

The database is attached as supplementary Information.

## References

[1] Al Salmi I, Hannawi S (2020). Birth weight is inversely correlated with blood pressure: A population-based study. J. Hypertens..

[2] Knop MR, Geng TT, Gorny AW, Ding R, Li C, Ley SH, Huang T (2018). Birth weight and risk of type 2 diabetes mellitus, cardiovascular disease, and hypertension in adults: A meta-analysis of 7,646,267 participants from 135 studies. J. Am. Heart Assoc..

[3] Li X, Yang R, Yang W, Xu H, Song R, Qi X, Xu W (2021). Association of low birth weight with cardiometabolic diseases in Swedish twins: A population-based cohort study. BMJ Open.

[4] Main KM, Jensen RB, Asklund C, Hoi-Hansen CE, Skakkebaek NE (2006). Low birth weight and male reproductive function. Horm. Res..

[5] Sadrzadeh S, Hui EVH, Schoonmade LJ, Painter RC, Lambalk CB (2017). Birthweight and PCOS: systematic review and meta-analysis. Hum. Reprod. Open.

[6] Juul F, Chang VW, Brar P, Parekh N (2017). Birth weight, early life weight gain and age at menarche: A systematic review of longitudinal studies. Obes. Rev..

[7] Faure C, Dupont C, Chavatte-Palmer P, Gautier B, Levy R, ALIFERT Collaborative Group (2015). Are semen parameters related to birth weight?. Fertil. Steril..

[8] Workman M, Kelly K (2017). Heavier birth weight associated with taller height but not age at menarche in US women born 1991–1998. Am. J. Hum. Biol..

[9] Evensen E, Emaus N, Kokkvoll A, Wilsgaard T, Furberg AS, Skeie G (2017). The relation between birthweight, childhood body mass index, and overweight and obesity in late adolescence: A longitudinal cohort study from Norway, The Tromsø Study, Fit Futures. BMJ Open.

[10] Kramer MS (2000). Invited commentary: Association between restricted fetal growth and adult chronic disease: Is it causal? Is it important?. Am. J. Epidemiol..

[11] Godfrey KM, Barker DJ (2001). Fetal programming and adult health. Public Health Nutr..

[12] Allvin K, Ankarberg-Lindgren C, Fors H, Dahlgren J (2008). Elevated serum levels of estradiol, dihydrotestosterone, and inhibin B in adult males born small for gestational age. J. Clin. Endocrinol. Metab..

[13] Martin WN, Pennell CE, Wang CA, Reynolds R (2020). Developmental programming and the hypothalamic–pituitary–adrenal axis. Curr. Opin. Endocr. Metab. Res..

[14] Martin-Estal I, De La Garza RG, Castilla-Cortazar I (2016). Intrauterine growth retardation (IUGR) as a novel condition of insulin-like growth factor-1 (IGF-1) deficiency. Rev. Physiol. Biochem. Pharmacol..

[15] Vaiserman AM (2018). Birth weight predicts aging trajectory: A hypothesis. Mech. Ageing Dev..

[16] Kaeberlein M, Rabinovitch PS, Martin GM (2015). Healthy aging: The ultimate preventative medicine. Science.

[17] Melzer D, Pilling LC, Ferrucci L (2020). The genetics of human ageing. Nat. Rev. Genet..

[18] Kirkwood TB (2005). Understanding the odd science of aging. Cell.

[19] Levine ME (2013). Modeling the rate of senescence: Can estimated biological age predict mortality more accurately than chronological age?. J. Gerontol. Ser. A Biomed. Sci. Med. Sci..

[20] Sebastiani P, Thyagarajan B, Sun F, Schupf N, Newman AB, Montano M, Perls TT (2017). Biomarker signatures of aging. Aging Cell.

[21] Kuzawa, C. W., Ryan, C. P., Adair, L. S., Lee, N. R., Carba, D. B., MacIsaac, J. L., et al. Birth weight and maternal energy status during pregnancy as predictors of epigenetic age acceleration in young adults from metropolitan Cebu, Philippines. *Epigenetics*. 1–11 (2022). 10.1080/15592294.2022.2070105 PMC9586628 35574972

[22] Quinn EB, Hsiao CJ, Maisha FM, Mulligan CJ (2023). Low birthweight is associated with epigenetic age acceleration in the first 3 years of life. Evol. Med. Public Health.

[23] Madden RA, McCartney DL, Walker RM, Hillary RF, Bermingham ML, Rawlik K (2021). Birth weight associations with DNA methylation differences in an adult population. Epigenetics.

[24] Slykerman RF, Joglekar MV, Hardikar AA, Satoor SN, Thompson JM, Jenkins A (2019). Maternal stress during pregnancy and small for gestational age birthweight are not associated with telomere length at 11 years of age. Gene.

[25] Abraham, C. R., Li, A. Aging-suppressor Klotho: Prospects in diagnostics and therapeutics. *Ageing Res. Rev.* 82, 101766 (2022). 10.1016/j.arr.2022.101766 36283617

[26] Kuro-o M, Matsumura Y, Aizawa H, Kawaguchi H, Suga T, Utsugi T (1997). Mutation of the mouse klotho gene leads to a syndrome resembling ageing. Nature.

[27] Pathare GV, Shalia KK (2019). Klotho: An emerging factor in neurodegenerative diseases. Biomed. Res. J..

[28] Drew DA, Katz R, Kritchevsky S, Ix JH, Shlipak MG, Newman AB (2021). Soluble Klotho and incident hypertension. Clin. J. Am. Soc. Nephrol..

[29] Cheng YW, Hung CC, Fang WH, Chen WL (2022). Association between soluble α-Klotho protein and metabolic syndrome in the adult population. Biomolecules.

[30] Typiak M, Piwkowska A (2021). Antiinflammatory actions of klotho: Implications for therapy of diabetic nephropathy. Int. J. Mol. Sci..

[31] Stamou MI, Colling C, Dichtel LE (2023). Adrenal aging and its effects on the stress response and immunosenescence. Maturitas.

[32] Ohlsson C, Labrie F, Barrett-Connor E, Karlsson MK, Ljunggren O, Vandenput L (2010). Low serum levels of dehydroepiandrosterone sulfate predict all-cause and cardiovascular mortality in elderly Swedish men. J. Clin. Endocrinol. Metab..

[33] Clark BJ, Prough RA, Klinge CM (2018). Mechanisms of action of dehydroepiandrosterone. Vitam. Horm..

[34] Hazeldine J, Arlt W, Lord JM (2010). Dehydroepiandrosterone as a regulator of immune cell function. J. Steroid Biochem. Mol. Biol..

[35] Schwartz AG (2022). Dehydroepiandrosterone, cancer, and aging. Aging Dis..

[36] Sato K, Iemitsu M (2018). The role of dehydroepiandrosterone (DHEA) in skeletal muscle. Vitam. Horm..

[37] Kirby DJ, Buchalter DB, Anil U, Leucht P (2020). DHEA in bone: The role in osteoporosis and fracture healing. Arch. Osteoporos..

[38] Gupta A, Saraf S, Kaur CD, Saraf S (2013). The potentials of dehydroepiandrosterone (DHEA) in dkin ageing process. Res. J. Top. Cosmet. Sci..

[39] Hildreth KL, Gozansky WS, Jankowski CM, Grigsby J, Wolfe P, Kohrt WM (2013). Association of serum dehydroepiandrosterone sulfate and cognition in older adults: Sex steroid, inflammatory, and metabolic mechanisms. Neuropsychology.

[40] Wu TT, Gao Y, Zheng YY, Ma YT, Xie X (2019). Association of endogenous DHEA/DHEAS with coronary heart disease: A systematic review and meta-analysis. Clin. Exp. Pharmacol. Physiol..

[41] Wang X, Feng H, Fan D, Zou G, Han Y, Liu L (2020). The influence of dehydroepiandrosterone (DHEA) on fasting plasma glucose, insulin levels and insulin resistance (HOMA-IR) index: A systematic review and dose-response meta-analysis of randomized controlled trials. Complement. Ther. Med..

[42] Camporez JPG, Akamine EH, Davel AP, Franci CR, Rossoni LV, de Oliveira Carvalho CR (2011). Dehydroepiandrosterone protects against oxidative stress-induced endothelial dysfunction in ovariectomized rats. J. Physiol..

[43] Masi AT, Rehman AA, Jorgenson LC, Smith JM, Aldag JC (2015). Sexual dimorphisms of adrenal steroids, sex hormones, and immunological biomarkers and possible risk factors for developing rheumatoid arthritis. Int. J. Endocrinol..

[44] Rigoulet M, Yoboue ED, Devin A (2011). Mitochondrial ROS generation and its regulation: Mechanisms involved in H_2_O_2_ signaling. Antioxid. Redox Signal..

[45] Hajam YA, Rani R, Ganie SY, Sheikh TA, Javaid D, Qadri SS (2022). Oxidative stress in human pathology and aging: Molecular mechanisms and perspectives. Cells.

[46] Liguori I, Russo G, Curcio F, Bulli G, Aran L, Della-Morte D (2018). Oxidative stress, aging, and diseases. Clin. Interv. Aging.

[47] Dubois-Deruy E, Peugnet V, Turkieh A, Pinet F (2020). Oxidative stress in cardiovascular diseases. Antioxidants.

[48] Baierle M, Nascimento SN, Moro AM, Brucker N, Freitas F, Gauer B (2015). Relationship between inflammation and oxidative stress and cognitive decline in the institutionalized elderly. Oxid. Med. Cell. Longev..

[49] Yaribeygi H, Sathyapalan T, Atkin SL, Sahebkar A (2020). Molecular mechanisms linking oxidative stress and diabetes mellitus. Oxid. Med. Cell. Longev..

[50] Hayes JD, Dinkova-Kostova AT, Tew KD (2020). Oxidative stress in cancer. Cancer Cell.

[51] Belenguer-Varea Á, Tarazona-Santabalbina FJ, Avellana-Zaragoza JA, Martínez-Reig M, Mas-Bargues C, Inglés M (2020). Oxidative stress and exceptional human longevity: Systematic review. Free Radic. Biol. Med..

[52] Wang Z, Wang Y, Liu H, Che Y, Xu Y, E, L. (2015). Age-related variations of protein carbonyls in human saliva and plasma: Is saliva protein carbonyls an alternative biomarker of aging?. Age.

[53] Gubandru M, Margina D, Tsitsimpikou C, Goutzourelas N, Tsarouhas K, Ilie M (2013). Alzheimer’s disease treated patients showed different patterns for oxidative stress and inflammation markers. Food Chem. Toxicol..

[54] Megson IL, Haw SJ, Newby DE, Pell JP (2013). Association between exposure to environmental tobacco smoke and biomarkers of oxidative stress among patients hospitalized with acute myocardial infarction. PLoS ONE.

[55] Milne GL, Musiek ES, Morrow JD (2005). F2-isoprostanes as markers of oxidative stress in vivo: An overview. Biomarkers.

[56] Matsuda M, Shimomura I (2013). Increased oxidative stress in obesity: Implications for metabolic syndrome, diabetes, hypertension, dyslipidemia, atherosclerosis, and cancer. Obes. Res. Clin. Pract..

[57] Mure K, Yoshimura N, Hashimoto M, Muraki S, Oka H, Tanaka S (2015). Urinary 8-iso-prostaglandin F2α as a marker of metabolic risks in the general Japanese population: The ROAD study. Obesity.

[58] Gan W, Liu XL, Yu T, Zou YG, Li TT, Wang S (2018). Urinary 8-oxo-7, 8-dihydroguanosine as a potential biomarker of aging. Front. Aging Neurosci..

[59] Gan W, Nie B, Shi F, Xu XM, Qian JC, Takagi Y (2012). Age-dependent increases in the oxidative damage of DNA, RNA, and their metabolites in normal and senescence-accelerated mice analyzed by LC–MS/MS: Urinary 8-oxoguanosine as a novel biomarker of aging. Free Radic. Biol. Med..

[60] Nie B, Gan W, Shi F, Hu GX, Chen LG, Hayakawa H (2013). Age-dependent accumulation of 8-oxoguanine in the DNA and RNA in various rat tissues. Oxid. Med. Cell. Longev..

[61] Maciejczyk M, Nesterowicz M, Szulimowska J, Zalewska A (2022). Oxidation, glycation, and carbamylation of salivary biomolecules in healthy children, adults, and the elderly: Can saliva be used in the assessment of aging?. J. Inflamm. Res..

[62] Kryston TB, Georgiev AB, Pissis P, Georgakilas AG (2011). Role of oxidative stress and DNA damage in human carcinogenesis. Mutat. Res..

[63] Black CN, Bot M, Scheffer PG, Penninx BW (2016). Sociodemographic and lifestyle determinants of plasma oxidative stress markers 8-OHdG and F2-isoprostanes and associations with metabolic syndrome. Oxid. Med. Cell. Longev..

[64] Bailo PS, Martín EL, Calmarza P, Breva SM, Gómez AB, Giráldez AP (2022). The role of oxidative stress in neurodegenerative diseases and potential antioxidant therapies. Adv. Lab. Med..

[65] Lencel P, Magne D (2011). Inflammaging: The driving force in osteoporosis?. Med. Hypotheses.

[66] De Martinis M, Franceschi C, Monti D, Ginaldi L (2006). Inflammation markers predicting frailty and mortality in the elderly. Exp. Mol. Pathol..

[67] Franceschi C, Garagnani P, Parini P, Giuliani C, Santoro A (2018). Inflammaging: A new immune–metabolic viewpoint for age-related diseases. Nat. Rev. Endocrinol..

[68] Ansar W, Ghosh S (2013). C-reactive protein and the biology of disease. Immunol. Res..

[69] Holmes C (2013). Systemic inflammation and Alzheimer's disease. Neuropathol. Appl. Neurobiol..

[70] Santos-Moreno P, Burgos-Angulo G, Martinez-Ceballos MA, Pizano A, Echeverri D, Bautista-Niño PK (2021). Inflammaging as a link between autoimmunity and cardiovascular disease: The case of rheumatoid arthritis. RMD Open.

[71] Moss SF, Blaser MJ (2005). Mechanisms of disease: Inflammation and the origins of cancer. Nat. Clin. Pract. Oncol..

[72] Jennings BJ, Ozanne SE, Dorling MW, Hales CN (1999). Early growth determines longevity in male rats and may be related to telomere shortening in the kidney. FEBS Lett..

[73] Luyckx VA, Compston CA, Simmen T, Mueller TF (2009). Accelerated senescence in kidneys of low-birth-weight rats after catch-up growth. Am. J. Physiol. Ren. Physiol..

[74] Tarry-Adkins JL, Chen JH, Smith NS, Jones RH, Cherif H, Ozanne SE (2009). Poor maternal nutrition followed by accelerated postnatal growth leads to telomere shortening and increased markers of cell senescence in rat islets. FASEB J..

[75] Tarry-Adkins JL, Martin-Gronert MS, Chen JH, Cripps RL, Ozanne SE (2008). Maternal diet influences DNA damage, aortic telomere length, oxidative stress, and antioxidant defense capacity in rats. FASEB J..

[76] Tarik, M., Ramakrishnan, L., Sinha, S., Sachdev, H. P. S., Tandon, N., Roy, A., Bhargava, S. K. The relationship of birth size and postnatal growth with cellular senescence in adults: Data from the New Delhi Birth Cohort. *Indian J. Pediatr.* 1–7 (2022). 10.1007/s12098-022-04174-4 35704216

[77] Kajantie E, Pietiläinen KH, Wehkalampi K, Kananen L, Räikkönen K, Rissanen A (2012). No association between body size at birth and leucocyte telomere length in adult life—Evidence from three cohort studies. Int. J. Epidemiol..

[78] Tarik M, Ramakrishnan L, Sinha S, Sachdev HPS, Tandon N, Roy A, Bhargava SK (2019). Association of birth outcomes and postnatal growth with adult leukocyte telomere length: Data from New Delhi birth cohort. Matern. Child Nutr..

[79] Masterson EE, Hayes MG, Kuzawa CW, Lee NR, Eisenberg DT (2019). Early life growth and adult telomere length in a Filipino cohort study. Am. J. Hum. Biol..

[80] Buxton JL, Walters RG, Visvikis-Siest S, Meyre D, Froguel P, Blakemore AI (2011). Childhood obesity is associated with shorter leukocyte telomere length. J. Clin. Endocrinol. Metab..

[81] Clemente DB, Maitre L, Bustamante M, Chatzi L, Roumeliotaki T, Fossati S (2019). Obesity is associated with shorter telomeres in 8 year-old children. Sci. Rep..

[82] Danese A, Pariante CM, Caspi A, Taylor A, Poulton R (2007). Childhood maltreatment predicts adult inflammation in a life-course study. Proc. Natl. Acad. Sci..

[83] McDade TW, Rutherford J, Adair L, Kuzawa CW (2010). Early origins of inflammation: Microbial exposures in infancy predict lower levels of C-reactive protein in adulthood. Proc. R. Soc. B Biol. Sci..

[84] Skilton MR, Viikari JS, Juonala M, Laitinen T, Lehtimäki T, Taittonen L (2011). Fetal growth and preterm birth influence cardiovascular risk factors and arterial health in young adults: The Cardiovascular Risk in Young Finns Study. Arterioscler. Thromb. Vasc. Biol..

[85] Tzoulaki I, Jarvelin MR, Hartikainen AL, Leinonen M, Pouta A, Paldanius M (2008). Size at birth, weight gain over the life course, and low-grade inflammation in young adulthood: Northern Finland 1966 Birth Cohort study. Eur. Heart J..

[86] Sattar N, McConnachie A, O’Reilly D, Upton MN, Greer IA, Smith GD, Watt G (2004). Inverse association between birth weight and C-reactive protein concentrations in the MIDSPAN Family Study. Arterioscler. Thromb. Vasc. Biol..

[87] Kofler T, Bossard M, Aeschbacher S, Tabord A, Ruperti Repilado FJ, van der Lely S (2016). The interrelationships of birthweight, inflammation and body composition in healthy adults. Eur. J. Clin. Investig..

[88] Goosby BJ, Cheadle JE, McDade T (2016). Birth weight, early life course BMI, and body size change: Chains of risk to adult inflammation?. Soc. Sci. Med..

[89] McDade TW, Koning SM (2021). Early origins of socioeconomic inequalities in chronic inflammation: Evaluating the contributions of low birth weight and short breastfeeding. Soc. Sci. Med..

[90] Carmeli C, Steen J, Petrovic D, Lepage B, Delpierre C, Kelly-Irving M (2020). Mechanisms of life-course socioeconomic inequalities in adult systemic inflammation: Findings from two cohort studies. Soc. Sci. Med..

[91] Bertran N, Camps J, Fernandez-Ballart J, Arija V, Ferre N, Tous M (2005). Diet and lifestyle are associated with serum C-reactive protein concentrations in a population-based study. J. Lab. Clin. Med..

[92] deRosset L, Strutz KL (2015). Developmental origins of chronic inflammation: A review of the relationship between birth weight and C-reactive protein. Ann. Epidemiol..

[93] Iñiguez G, Gallardo P, Castro JJ, Gonzalez R, Garcia M, Kakarieka E (2019). Klotho gene and protein in human placentas according to birth weight and gestational age. Front. Endocrinol..

[94] Żelaźniewicz A, Nowak-Kornicka J, Pawłowski B (2022). S-Klotho level and physiological markers of cardiometabolic risk in healthy adult men. Aging (Albany NY).

[95] Brandenburg VM, Kleber ME, Vervloet MG, Larsson TE, Tomaschitz A, Pilz S (2015). Soluble klotho and mortality: The Ludwigshafen risk and cardiovascular health study. Atherosclerosis.

[96] Liang WY, Wang LH, Wei JH, Li QL, Li QY, Liang Q (2021). No significant association of serum klotho concentration with blood pressure and pulse wave velocity in a Chinese population. Sci. Rep..

[97] Seiler S, Wen M, Roth HJ, Fehrenz M, Flügge F, Herath E (2013). Plasma Klotho is not related to kidney function and does not predict adverse outcome in patients with chronic kidney disease. Kidney Int..

[98] Amaro-Gahete FJ, Jurado-Fasoli L, Sanchez-Delgado G, García-Lario JV, Castillo MJ, Ruiz JR (2020). Relationship between plasma S-Klotho and cardiometabolic risk in sedentary adults. Aging (Albany NY).

[99] Orces, C. H. The association between metabolic syndrome and the anti-aging humoral factor klotho in middle-aged and older adults. *Diabetes Metab. Syndr. Clin. Res. Rev.* 102522 (2022). 10.1016/j.dsx.2022.102522 35660935

[100] Dote-Montero M, Amaro-Gahete FJ, Jurado-Fasoli L, Gutierrez A, Castillo MJ (2019). Study of the association of DHEAS, testosterone and cortisol with S-Klotho plasma levels in healthy sedentary middle-aged adults. Exp. Gerontol..

[101] Gavin KM, Bessesen DH (2020). Sex differences in adipose tissue function. Endocrinol. Metab. Clin..

[102] Lee J, Kim D, Lee HJ, Choi JY, Min JY, Min KB (2022). Association between serum klotho levels and cardiovascular disease risk factors in older adults. BMC Cardiovasc. Disord..

[103] Jurado-Fasoli L, Amaro-Gahete FJ, Gutiérrez Á, Castillo MJ (2019). Alcohol consumption and S-Klotho plasma levels in sedentary healthy middle-aged adults: A cross sectional study. Drug Alcohol Depend..

[104] Jurado-Fasoli L, Amaro-Gahete FJ, Arias-Tellez MJ, Gil A, Labayen I, Ruiz JR (2021). Relationship between dietary factors and S-Klotho plasma levels in young sedentary healthy adults. Mech. Ageing Dev..

[105] Quintero-Platt G, González-Reimers E, Rodríguez-Gaspar M, Martín-González C, Pérez-Hernández O, Romero-Acevedo L (2017). Alpha Klotho and fibroblast growth factor-23 among alcoholics. Alcohol Alcohol..

[106] Inci A, Sari F, Coban M, Olmaz R, Dolu S, Sarıkaya M, Yılmaz N (2016). Soluble Klotho and fibroblast growth factor 23 levels in diabetic nephropathy with different stages of albuminuria. J. Investig. Med..

[107] Sze L, Bernays RL, Zwimpfer C, Wiesli P, Brändle M, Schmid C (2012). Excessively high soluble Klotho in patients with acromegaly. J. Intern. Med..

[108] Sari F, Gumuslu S, Cetinkaya R, Sarikaya M, Yalcin AD (2017). High serum soluble CD200 levels in patients with autosomal dominant polycystic kidney disease. J. Investig. Med..

[109] Alkalbani M, Prabhu G, Lagbo J, Qayyum R (2022). Serum Klotho and pulse pressure; insight from NHANES. Int. J. Cardiol..

[110] Wu SE, Chen WL (2022). Soluble klotho as an effective biomarker to characterize inflammatory states. Ann. Med..

[111] Martín-Núñez E, Donate-Correa J, Ferri C, López-Castillo Á, Delgado-Molinos A, Hernández-Carballo C (2020). Association between serum levels of Klotho and inflammatory cytokines in cardiovascular disease: A case-control study. Aging (Albany NY).

[112] Szathmari M, Reusz G, Tulassay T (2000). Low birth weight, adrenal and sex hormones and their correlation with carbohydrate metabolism and cardiovascular physiology, investigated in young adulthood. Orv. Hetil..

[113] Santos-Silva R, Fontoura M, Guimarães JT, Barros H, Santos AC (2022). Association of dehydroepiandrosterone sulfate, birth size, adiposity and cardiometabolic risk factors in 7-year-old children. Pediatr. Res..

[114] Luo ZC, Fraser WD, Julien P, Deal CL, Audibert F, Smith GN (2006). Tracing the origins of “fetal origins” of adult diseases: Programming by oxidative stress?. Med. Hypotheses.

[115] Thompson, L. P., Al-Hasan, Y. Impact of oxidative stress in fetal programming. *J. Pregnancy,* **2012** (2012). 10.1155/2012/582748 PMC3403156 22848830

[116] Chiavaroli V, Giannini C, D'Adamo E, de Giorgis T, Chiarelli F, Mohn A (2009). Insulin resistance and oxidative stress in children born small and large for gestational age. Pediatrics.

[117] Mohn A, Chiavaroli V, Cerruto M, Blasetti A, Giannini C, Bucciarelli T, Chiarelli F (2007). Increased oxidative stress in prepubertal children born small for gestational age. J. Clin. Endocrinol. Metab..

[118] Ojeda NB, Grigore D, Robertson EB, Alexander BT (2007). Estrogen protects against increased blood pressure in postpubertal female growth restricted offspring. Hypertension.

[119] Alexander BT, Dasinger JH, Intapad S (2014). Effect of low birth weight on women’s health. Clin. Ther..

[120] Franco MC, Kawamoto EM, Gorjão R, Rastelli VM, Curi R, Scavone C (2007). Biomarkers of oxidative stress and antioxidant status in children born small for gestational age: Evidence of lipid peroxidation. Pediatr. Res..

[121] Yeap BB, Araujo AB, Wittert GA (2012). Do low testosterone levels contribute to ill-health during male ageing?. Crit. Rev. Clin. Lab. Sci..

[122] Gorelik SG, Belousova ON, Treneva EV, Bulgakova SV, Zakharova NO, Nesterenko SA (2022). Effect of daily rhythms of cortisol secretion on the rate of aging in men. Arch. Razi Inst..

[123] Lucas A, Fewtrell MS, Cole TJ (1999). Fetal origins of adult disease-the hypothesis revisited. BMJ.

[124] Källén B (1995). A birth weight for gestational age standard based on data in the Swedish Medical Birth Registry, 1985–1989. Eur. J. Epidemiol..

[125] Gangestad SW, Merriman LA, Thompson ME (2010). Men’s oxidative stress, fluctuating asymmetry and physical attractiveness. Anim. Behav..

[126] Faul F, Erdfelder E, Lang A-G, Buchner A (2007). G*Power 3: A flexible statistical power analysis program for the social, behavioral, and biomedical sciences. Behav. Res. Methods.

